# Cockroaches as an Emerging Invertebrate Model in Bioscience Research: *Gromphadorhina portentosa*

**DOI:** 10.3390/ani16111644

**Published:** 2026-05-27

**Authors:** Maria Vittoria Varoni, Filomena Dessì, Elena Baralla, Pier Andrea Serra, Gaia Rocchitta, Ana Maria Molina Lopez

**Affiliations:** 1 Department of Veterinary Medicine, University of Sassari, 07100 Sassari, Italy; varoni@uniss.it (M.V.V.); 94.filo@gmail.com (F.D.); 2 Department of Medicine, Surgery and Pharmacy, University of Sassari, 07100 Sassari, Italy; paserra@uniss.it (P.A.S.); grocchitta@uniss.it (G.R.); 3 Department of Anatomía y Anatomía Patológica Comparadas, y Toxicología, UIC Zoonosis y Enfermedades Emergentes, Universidad de Córdoba, 14071 Córdoba, Spain; ft2moloa@uco.es

**Keywords:** cockroaches, *Gromphadorhina portentosa*, alternative model, invertebrates, bioscience research

## Abstract

The increasing social, ethical and regulatory pressure on the use of vertebrates in bioscience research has promoted the development of alternative models. The use of invertebrate models is very common in basic research, where insects are used in many studies. Cockroaches such as *Gromphadorhina portentosa* have emerged as a promising model organism due to their ease of maintenance, breeding in laboratory conditions, longevity, physiological resilience, size, and ability to be manipulated for sample collection. Similarly, prior to 2010, the *Periplaneta americana* was widely used as a bioscience model and shares many of the attributes of the *Gromphadorhina portentosa*. The objective of our study was to carry out a review in which we described the biological characteristics and analyzed the application of cockroaches, and specifically *Gromphadorhina portentosa*, as an alternative model in bioscience research. To do so, we established the period from 2010 to 2025 to carry out the bibliographic search in international scientific databases, PubMed and Scopus, using only peer-reviewed scientific publications on cockroach applications in bioscience research. This review outlines the principal biological characteristics of cockroaches and evaluates their suitability as experimental models. Additionally, potential humane euthanasia methods are discussed. The most common applications of cockroaches in laboratory experimentation are described, and a critical analysis of their advantages and limitations as model organisms is presented. The analysis reveals significant potential for the use of cockroaches in preliminary and educational studies, as well as an opportunity to expand the diversity of species in scientific research, applying the principle of replacement of the use of animals in experimentation. The document highlights the need for protocol standardization and regulatory recognition for this species, offering an updated perspective on the state of the art regarding its rational incorporation into bioscience research.

## Simple Summary

The use of animals in scientific research is strongly debated due to ethical concerns and stricter regulations, especially when vertebrates are involved. This has led the scientific community to search for alternative models in order to reduce animal use while still providing reliable results. In this context, cockroaches, and particularly *Gromphadorhina portentosa* (commonly known as the Madagascar hissing cockroach), are gaining attention as promising emerging invertebrate models. They are particularly suitable for experimental studies because they are easy to maintain in the laboratory, able to reproduce efficiently and have relatively long lives. In this review, we analyzed scientific studies published between 2010 and 2025 to evaluate the potential of cockroaches as alternative models in bioscience research. We describe their main biological features, summarize how they are currently used in experiments, and discuss their advantages and limitations. We also address humane methods for ending their life in research settings. Our findings suggest that cockroaches could play an important role in scientific and educational research, helping to reduce the use of vertebrate animals. However, further work is needed to standardize experimental methods and to achieve wider regulatory acceptance of these organisms.

## 1. Introduction

The use of animals in biomedical research has been fundamental for advancing knowledge in physiology, pharmacology, neuroscience, and toxicology, among others. Animal models such as rodents, rabbits, and fish have enabled the validation of biological hypotheses, the development of clinical therapies, and the assessment of health and environmental risks [1,2,3,4]. However, this approach has raised increasing ethical and social concerns regarding animal welfare, leading to significant regulatory reforms. In the European Union, Directive 2010/63/EU [5] establishes that the use of alternative methods must be prioritized before resorting to vertebrates in research.

In response to these demands, the principles of the 3Rs (replacement, reduction, and refinement) have been consolidated as pillars of the ethical use of animals in science. Organizations such as the Federation of European Laboratory Animal Science Associations (FELASA) and the European Animal Research Association (EARA) align with the broader European framework that promotes the use of alternative models whenever scientifically feasible [6].

Among invertebrates, classical models such as the fruit fly (*Drosophila melanogaster*, Meigen, 1830) and the silkworm (*Bombyx mori*, Linnaeus, 1758) have been widely used due to their ease of breeding, the development of genetic tools, and their relevance in molecular studies. However, these species present significant limitations: size, difficulty in extracting tissues or biological fluids, and limited utility in long-term toxicological studies or experiments requiring repeated manipulation [7,8,9]. The physiology and biochemistry of invertebrates provide opportunities for innovative approaches that have yet to be fully explored. Strategic investments in invertebrate model organisms have led to significant advancements in fundamental understanding of living systems, health, and disease, while also offering technological tools that drive progress in bioscience research [10].

Among invertebrates, cockroaches are increasingly used as models in biomedical [11], toxicological [12], and robotics [13] research due to their ease of maintenance, relatively large body size, low cost, and remarkable adaptability, which make them suitable for a wide range of experimental applications. The widespread presence of cockroaches in exhibitions, homes, and scientific settings contributes to their diverse context. This includes studies of their interactions with humans and the potential diseases they transmit. Recent years have seen a significant increase in the use of these species as experimental models for analyzing nociception and physiological responses to stress [14,15,16]. The current research landscape also places particular emphasis on developing shared strategies for anesthesia and euthanasia, aiming to minimize suffering while ensuring an ethical, scientifically rigorous, and easily replicable approach [17].

*Gromphadorhina portentosa* (Schaum, 1853), commonly known as the Madagascar hissing cockroach, is a large cockroach native to Madagascar that has begun to gain prominence over the past decade as a versatile and ethically acceptable alternative model. Its long lifespan (up to 5 years), docile behavior, environmental resilience, absence of functional wings compared with many pest cockroach species and ease of maintenance without specialized facilities make it a suitable species for multiple applications [18,19,20]. Its body size allows for the extraction of hemolymph, direct observation of physiological processes, and exposure to chemical compounds through various administration routes (oral, topical, aerial, injection) with the option of individualized dose control [21,22]. In addition, their widespread availability through commercial breeding sources has contributed to their increasing use in experimental settings.

Before the increasing use of *G. portentosa*, the American cockroach (*Periplaneta americana*, Linnaeus, 1758) was a model organism used in experimental studies in several fields [23,24] such as neurophysiology [25], toxicology (in addressing toxicological mechanisms as well as a sensor of environmental pollution) [12], metabolic regulation, social behavior, neurophysiology, endocrinology [24], and legged locomotion [26,27]. Both cockroaches are inexpensive to maintain, easy to handle because of their relatively large size, and highly prolific, making them simple to breed in large numbers. These characteristics, together with their widespread availability, make them excellent model organisms for a wide range of experimental studies.

*G. portentosa* presents both advantages and disadvantages compared with *P. americana*. Its larger body size, slower movement, and inability to fly make it particularly well suited for handling and experimental manipulations. Moreover, *G. portentosa* produces hissing sounds that enable research into communication and defense mechanisms [28]. They are more widely accepted in educational environments [29,30], as they are classified as an exotic species rather than an invasive pest like *P. americana*. However, *G. portentosa* has a longer life cycle than *P. americana* and fewer generational cycles.

*P. americana* requires handling by expert personnel and the implementation of stringent biosecurity, being a very fast and invasive pest species. Moreover, it may induce allergic sensitization among operators.

Actually, the use of cockroaches is not regulated by European animal welfare directives and is not included in Directive 2010/63/EU [5]. Ethical authorization is not required for its use in research in most European countries at the moment. FELASA and EARA support the use of invertebrates as alternative models within the framework of the 3Rs. This is particularly encouraged in contexts where replacement of vertebrates is scientifically feasible, such as preliminary research or training and educational activities.

To establish this cockroach as an alternative model, studies on its biology, physiology, and behavior are necessary so that it can be standardized as a model organism. This article aims to provide a critical and comprehensive review of the use of cockroaches, focusing where possible on *G. portentosa* as a model organism in bioscience research. It addresses its key biological characteristics, main application areas, and types of studies in which it has been used.

## 2. Methodology

With this aim, we conducted a narrative review of the use of cockroaches as an alternative model in bioscience research, using the international scientific databases PubMed and Scopus. This procedure allowed for the selection of literature published between 2010 and 2025, in accordance with the need to cover the most recent and relevant contributions in these disciplines. Selected publications included peer-reviewed original research articles, review papers, and relevant books or book chapters published in English. Duplicate publications, conference abstracts, and non-peer-reviewed publications were excluded. Seminal or foundational studies published outside this period (2010–2025) were exceptionally included where they provided essential background information. Additionally, regulatory and technical documents from official organizations were reviewed, including the EARA, the FELASA, and Directive 2010/63/EU of the European Parliament. A total of 83 studies met eligibility criteria and were incorporated in this review.

## 3. Cockroaches’ General Characteristics

Cockroaches are insects classified within the class Insecta and order *Blattodea*. Currently, about 4600 species of cockroaches have been identified, and over 460 genera are described in the world. They can be divided into six families—*Cryptocercidae*, *Blattidae*, *Blattellidae*, *Blaberidae*, *Corydidae* and *Ectobiidae*—and are widespread in almost all habitats, from tropical and temperate forests to grasslands, steppes, swamps, coastal areas, and deserts [31]. They play a crucial role in ecosystems, acting primarily as decomposers. They feed on decaying organic matter, such as animal and plant remains. They can act as herbivores, feeding on young vegetation, as predators of other insects, and often serve as a source of food for numerous groups of invertebrates and vertebrates [32]. The life cycle of cockroaches is divided into three main stages, egg, nymph, and adult, representing a key aspect of their extraordinary ability to adapt [32]. They are heterometabolous insects, meaning they progress from the nymph stage to the adult stage through multiple molts. The duration and number of molts vary between different species and even between individuals of the same species [32,33,34].

Generally, their ability to reproduce rapidly and in large numbers allows them to optimize the resources available in their environment. Different main reproductive strategies can be found in the order *Blattodea*: oviparous, ovoviviparous and viviparous [35].

Like all insects, cockroaches are characterized by three distinct body parts, or tagmata (head, thorax, and abdomen), three pairs of legs, compound eyes, and a pair of antennae. Like all cockroaches, they also have a pair of sensory appendages at the end of their abdomen, known as cerci. The legs are made up of: coxa, trochanter, femur, tibia, and tarsi; dorsal and ventral sclerites; and spiracles [36].

In insects, hemolymph is the major extracellular fluid constituting 15–75% of the volume of the insect depending on the species and the physiological state [37]. It plays a crucial role in the transport of nutrients and signaling molecules to different body compartments [38]. Insects’ hemolymph contains functional hemocytes (granulocytes, plasmatocytes, and oenocytes) that allow the study of immune processes such as phagocytosis, encapsulation, and antimicrobial peptide production [39]. It is a clear or pigmented liquid with a variable pH depending on the species. Its composition varies between insect orders. In the *Blattodea* order, the main hemolymph components are sodium and chloride, followed by other components like amino acids, potassium, calcium, and magnesium. Moreover, its composition can vary depending on physiological and environmental conditions [37]. Basseri et al. [40] reported that the injection of bacteria in the abdominal cavity of *Periplaneta americana* induced a hemolymph response producing peptides to combat against Gram-positive bacteria. It is important to note that some studies on insect immunity, such as those conducted on *G. portentosa*, have revealed immune responses to toxins and pathogens. These species have similar hemocytic physiology, allowing for partial extrapolation of the results in immunotoxicological contexts [21].

### 3.1. Gromphadorhina portentosa

*G. portentosa*, the Madagascar hissing cockroach, is a species belonging to the order *Blattodea* and the family *Blaberidae*. It has been introduced as a model species in biomedical and toxicological research due to its robustness, longevity, docile behavior, being handled without anesthesia, and ease of maintenance and breeding under laboratory conditions [12].

**Figure 1:**
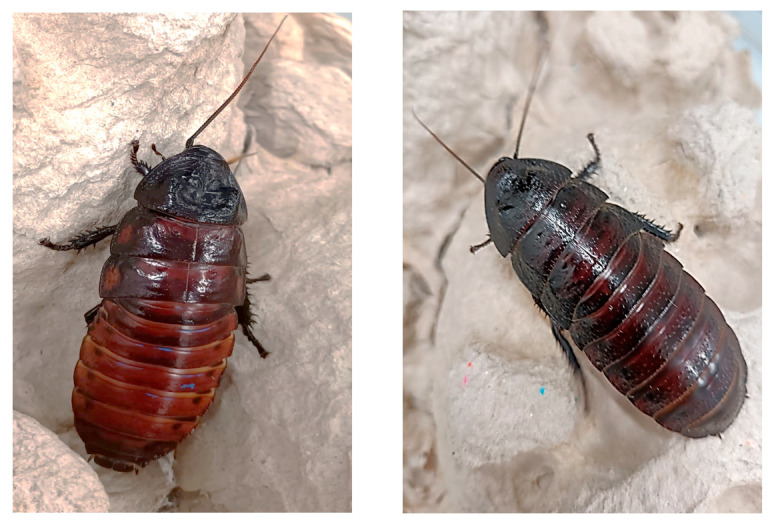
Male (**left**) and female (**right**) *Gromphadorhina portentosa* specimens (images from the breeding colony of the University of Sassari, Italy).

**Figure 2:**
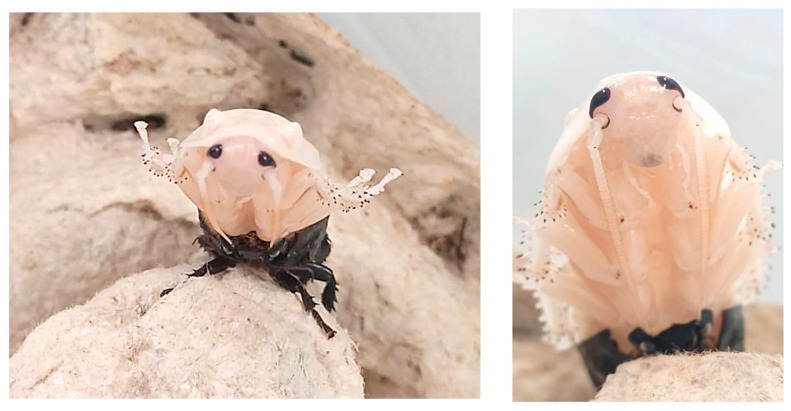
*Gromphadorhina portentosa* specimen while molting (images from the breeding colony of the University of Sassari, Italy).

**Figure 3:**
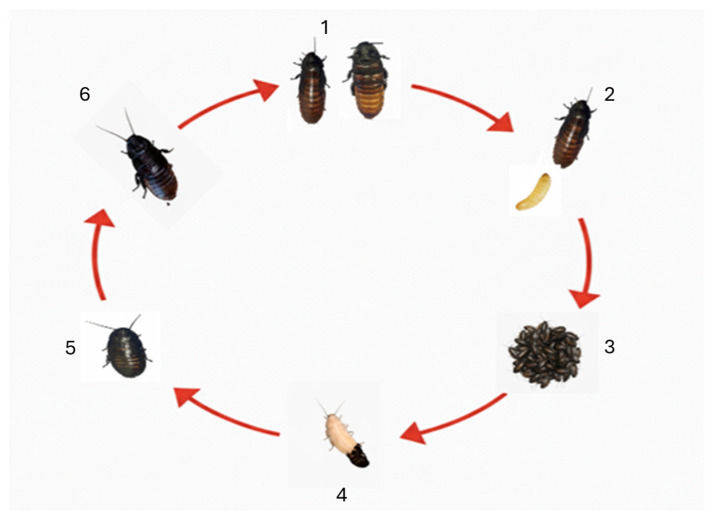
Life cycle of *Gromphadorhina portentosa*: (1) adults, (2) ootheca, (3) nymphs, (4) nymph while molting (5) and (6) nymphs as miniature adult.

Adults reach a length of between 6 and 10 cm (Figure 1), which facilitates handling without the need for specialized tools. They have a 2–5-year lifespan and develop through incomplete metamorphosis, progressing through three primary stages: eggs, nymphs that resemble miniature adults, and adults. They are ovoviviparous, with females giving birth to 15–60 live nymphs after the eggs develop inside the ootheca. The gestation period in laboratory conditions is approximately 60 days. The nymphs, measuring about 6–8 mm at birth, grow through several molting stages until they reach adulthood and sexual maturity in approximately six months (Figure 2). They exhibit characteristic sexual dimorphism: males have thoracic protrusions and have feather-like antennae [36]. Whistling sounds are produced through the spiracles by both sexes when threatened or disturbed, but they are also used by males during courtship, mating, and in intraspecific fights for territory. Their life cycle (Figure 3) can exceed four years in captivity; this fact could allow for chronic, reproductive and developmental toxicity studies [12,21].

This species does not require complex laboratory animal facilities or strict conditions: it is maintained at room temperature (24–28 °C), with moderate humidity (60–70%) and a 12 h/12 h inverted light/dark cycle, and fed with fruits, vegetables, and dry feed such as pet food. Additionally, its gregarious behavior, large size, inability to climb smooth surfaces, and resistance to environmental conditions make it ideal for laboratories with limited resources [21,41].

#### Behavior

The characteristic behavior of *G. portentosa*, such as its ability to produce “hissing” sounds by expelling air through its spiracles, is closely linked to the structure and function of its central nervous system. This behavior has been studied by Parker et al. [42], who analyzed the acoustic properties of defensive hisses following exposure to the hormone 20-hydroxyecdysone. The results showed significant modulation in the frequency and intensity of the sounds, suggesting a functional shift from defensive to aggressive signaling, mediated by endocrine mechanisms.

The cockroach’s head houses the brain, which regulates its bodily functions and behavior. The central nervous system of *G. portentosa* features a compact brain, a ventral chain of segmented ganglia, and accessible thoracic nerves. Thoracic ganglia play a key role in integrating sensory and motor signals. Regarding accessible thoracic nerves, studies on insect neuroanatomy suggest that thoracic ganglia contain neuronal networks responsible for motor activity [43] which reinforces the hypothesis of a hierarchical functional distribution within the central nervous system of arthropods. This model proved to be useful for studying the neurochemistry of the central complex, a key region involved in motor coordination and sensory integration [44]. On the other hand, regarding evasive behavior, Ou and Cleland [45] documented the escape strategies in response to looming visual stimuli and localized thermal cues. The individuals exhibited organized directional responses, contradicting previous studies that suggested random behavior. This finding reinforces the idea that *G. portentosa* possesses integrated sensory capabilities that allow for contextual evaluation of danger. Other authors such as Li et al. [46] investigated the biomechanical strategies employed by various *Blattodea* species to restore ventral orientation after being positioned supine, a condition that poses a significant threat to their mobility and ecological viability. Specifically, the authors report that *G. portentosa*, compared to other cockroaches examined, utilizes structural torsion of the exoskeleton, complemented by the coordinated action of its locomotor appendages, to generate the necessary torque for self-righting. These findings highlight a functional convergence in self-righting patterns, underpinned by adaptive morphological and behavioral modifications that enhance the biomechanical efficiency of the maneuver.

Overall, the structure of *G. portentosa*’s nervous system not only enables complex defensive and communicative behaviors but also makes it a valuable organism for scientific studies on sensory integration, motor coordination, and brain activity under experimental conditions. Consequently, these studies describe *G. portentosa* as a robust experimental model for investigating invertebrate behavior, with applications in neuroethology, endocrinology, and sensory physiology [43,44,46].

### 3.2. Euthanasia

Euthanasia is a critical point in animal experimentation. The euthanasia method must be easy to perform, rapid, and effective. There are multiple euthanasia methods available, but not all can be applied to every animal model, with specific recommendations depending on the case [5]. Similarly, in order to standardize *G. portentosa* as an alternative model, it is essential to establish the most appropriate euthanasia protocol, ensuring minimal stress during its execution. This safeguards welfare and prevents potential alterations in experimental results due to the euthanasia method used. Tucker et al. [17] conducted a study on euthanasia methods in four cockroach species: Dubia (*Blaptica dubia*, Serville, 1838), red runner (*Shelfordella lateralis*, Walker, 1868), Madagascar hissing (*Gromphadorhina portentosa*), and giant cave (*Blaberus giganteus*, Linnaeus, 1758), considering the loss of movement as death (irreversible loss of consciousness). After their study, they concluded that cockroach species may be euthanized through prolonged exposure (24 h) to isoflurane, or by isoflurane anesthesia followed by brief immersion (≤15 min) in 70% isopropyl alcohol. On the other hand, Barnes et al. [47] evaluated in cave cockroaches (*Blaberus giganteus*) and Madagascar hissing cockroaches (*Gromphadorhina portentosa*) the use of soapy water and freezing as secondary steps in euthanasia following prior anesthetic induction with CO_2_, isoflurane, or a combination of both. These authors reported that the use of 5% soapy water and freezing at −80 °C respectively were highly effective as secondary euthanasia methods in groups of animals, although they recommend using maceration of these insects as the final confirmation method.

## 4. Bioscience Research Applications of Cockroaches

**Figure 4:**
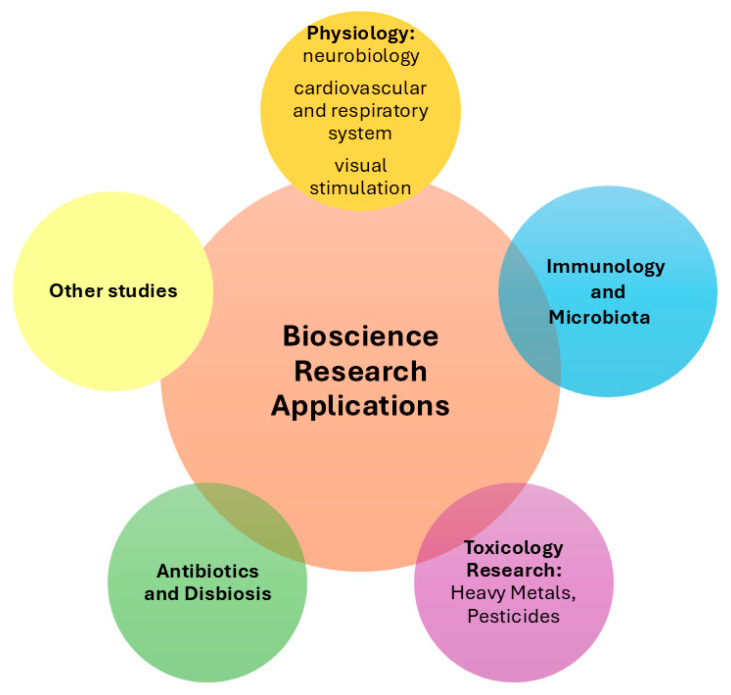
Cockroaches’ main bioscience research applications.

Although cockroaches have traditionally been used in educational and scientific outreach contexts, various recent experimental studies have demonstrated their potential as a model organism in biomedical research (Figure 4). Their morphology, physiology, and experimental tolerance make them a suitable system for analyzing complex physiological responses, especially in neurophysiology and immunology [21,38,44]. Moreover, cockroaches have emerged as a very popular alternative animal model in several scientific disciplines like biochemistry, pharmacology, and toxicology because of their biochemical and physiological similarities to mammals.

### 4.1. Physiology Studies

Several biochemical and physiological similarities between cockroaches and mammals have been described, such as metabolic, immune, and neurotransmitter systems [48,49,50]. Cockroaches do not have a complex central nervous system, but they conserve several neurotransmitter patterns like the serotoninergic, cholinergic, GABAergic, and dopaminergic ones. Therefore, they have been used as an alternative animal model to study several pharmaceuticals’ effects [50]. Zhukovskaya et al. [51] evaluated how the ultraviolet and green visual channels affect the individual behavior of *P. americana*. They suggested that excitation of the green channel stimulates locomotor behavior, while sleep-like immobility behavior was triggered by low-intensity UV stimulation. They concluded that, probably, bright illumination caused stress, that UV and green visual channels mediated different visual functions and that both were mutually antagonistic.

#### Physiology Studies Using *Gromphadorhina portentosa* as an Alternative Animal Model

The size and characteristics of this experimental model have enabled the use of the stereotaxic procedure for implanting neurochemical sensors in the central complex (CX), allowing for the monitoring of dissolved O_2_ via telemetry in free-walking *G. portentosa* [44]. This study is highly significant and pioneering, as the CX plays key roles in motor control, navigation, sensory integration, attention, sleep regulation, and memory. Determining dissolved O_2_ for investigating the neurochemical response of insect neural cells to physicochemical stimulations or to the administration of different drugs will be essential to ensure the proper physiological status of the insect, thus enabling the development of a valid model for this type of research [44]. The study of the insect cardiovascular system is important, as the pulsations of insect and human hearts are regulated by similar mechanisms based on the depolarization of myocardial cells. Claros-Guzman et al. [38] used this model to study cardiac physiology, employing a new video recording method coupled with an isotonic transducer for the three-dimensional study of the heart and intracardiac valves, as well as their possible response to the application of different cholinergic neurotransmitters. They examined the relationship between intracardiac valve movement, hemolymph flow, diastole, and systole. Their results suggested a combined action of cholinergic agonists in the regulation of heart rate, intracardiac valve function, and the cardiac cycle. Streicher et al. [52] studied the relationship between the respiratory and cardiovascular systems by analyzing oxygen consumption and heart rate, correlating them with body size and temperature. These authors observed that body size influences metabolic rate and heart rate in different ways: as body size increases, metabolic rate rises, while heart rate decreases. However, these changes do not follow a simple linear relationship. Additionally, temperature similarly affects both variables. This connection between body size and temperature is likely due to their mutual dependence on similar metabolic processes and/or interconnected regulatory mechanisms.

### 4.2. Immunology and Microbiota

Basseri et al. [40] used the *P. americana* as an experimental animal model. They isolated an antimicrobial peptide to combat Gram-positive bacteria from the hemolymph 24 h after they had injected Gram-positive or Gram-negative bacteria into the abdominal cavity of two groups of cockroaches separately. They concluded that the *P. americana* has the ability to produce peptides to combat Gram-positive bacteria when an immune challenge is mounted.

The immune system of insects has been characterized by Browne et al. [39], who identified various types of hemocytes involved in phagocytosis, encapsulation, and melanization.

The studies reported above demonstrated that cockroaches’ hemolymph could react to bacterial challenges with a typical insect immune response, including phenoloxidase activation and antimicrobial peptide production. Similar studies have been conducted on other insects, such as yellow mealworm (*Tenebrio molitor* Linnaeus, 1758), which exhibit conserved immune responses [53].

Additionally, cockroaches’ intestinal microbiota, which is complex and sensitive to environmental contaminants, represents a promising field for research on dysbiosis and antimicrobial resistance [54]. It is worth noting that some additional studies on these topics have been conducted on German cockroaches (*Blattella germanica*, Linnaeus,1767), and although they do not directly correspond to *G. portentosa*, they exhibit relevant physiological similarities that allow extrapolations [20,55,56].

#### Immunology and Microbiota Studies Using *Gromphadorhina portentosa* as an Animal Model

Chua et al. [21] reported that *G. portentosa* can be used to study virulence, host–pathogen interactions, innate immune responses, and drug efficacy. They infected this cockroach with bacteria *Burkholderia mallei*, *B. pseudomallei*, and *B. thailandensis*. The virulence was compared with rodent models, concluding that the hissing cockroach is a viable surrogate animal model for the three tested *Burkholderia* species.

### 4.3. Toxicology Research

Experimental toxicology aims to identify the adverse effects of chemical compounds on living organisms. In this field, insects have increasingly been recognized as alternative models, particularly in ecotoxicological, neurotoxicology, and gut dysbiosis studies.

Cockroaches have been largely used to assess the toxicological impact of several emerging contaminants. Rodrigues et al. [14] exposed the cinereous cockroach (*Nauphoeta cinerea* Olivier, 1789) to a solution of HgCl_2_ (10, 20, and 40 mg L^−1^ in drinking water) for 7 days. After euthanasia, head samples were collected to assess oxidative stress. It was observed that as the exposure concentration increased, there was a dose-dependent rise in the insect’s mortality. Following the sample analysis, they reported that the activity of peroxidase and thioredoxin reductase was suppressed at 20 mg L^−1^ and 40 mg L^−1^ when compared to the control group; in addition, glutathione S-transferase (GST) activity showed a significant reduction compared to the control. These findings were associated with a reduction in GSH levels and an elevation in both hydroperoxide and thiobarbituric acid reactive substance (TBARS) production. In conclusion, they reported that mercury exposure can trigger indicators of oxidative stress in insects by affecting their survival rate and disrupting key antioxidant defense mechanisms.

Furthermore, Adedara et al. [57] exposed (*N. cinerea*) nymphs to MeHg at concentrations of 0, 0.03125, 0.0625, 0.125, 0.25, and 0.5 mg per g of feed for 35 days to evaluate their behavior and biochemical parameters. The authors observed a significant reduction in distance traveled, time spent moving, turn angle, and body rotation. They also reported a significant decrease in acetylcholinesterase activity and an increase in oxidative stress (decreased total GST activity and thiol levels, along with increased TBARS production and 2′,7′-dichlorofluorescein (DFCH) oxidation). Moreover, the same authors demonstrated the ability of luteolin, a polyphenolic compound of plant origin, to prevent oxidative stress and neurobehavioral deficits using the cockroach *N. cinerea* as a model of MeHg^+^ toxicity [58]. The results of the studies reported above are consistent, indicating that mercury exposure would affect not only adult forms but also nymphs. This was also confirmed by Piccoli et al. [49], who evaluated the effects after acute, intermediate, and chronic administration of MeHg^+^ in nymphs of *N. cinerea*. Piccoli et al. [49] concluded that the cockroach *N. cinerea* is a valuable animal model for translational research, before subsequent research in vertebrates in accordance with the 3R principles. They, in fact, highlight the overlap of results obtained in this study and their previous results observed in rodents and mammals.

Kaur and Rawal [59] conducted a systematic review on different model organisms, including mealworm beetle (*Tenebrio molitor*), superworm beetle (*Zophobas atratus*, Fabricius 1775), and *Blattella germanica*, exposed to arsenic, cadmium, lead, and mercury. They highlighted how the intestinal microbiota of insects with comparable digestive physiology was sensible to heavy metals. Severe intestinal dysbiosis was identified. Most of these studies were subchronic (2–6 weeks).

Moreover, cockroaches are widely used in ecotoxicology studies [12,22,60]. Exposure to atrazine (herbicide), alone or in combination with ciprofloxacin (fluoroquinolone antibiotic), in the cinereous cockroach (*Nauphoeta cinerea*) was studied by Adedara et al. [60]. Terrestrial organisms may be inadvertently exposed, as the detection of atrazine and ciprofloxacin in soil is associated with intensive anthropogenic agricultural activities and unintentional discharge of industrial waste into the environment. For this reason, the authors decided to conduct a study of single and combined exposure to both compounds to deepen knowledge about the toxicological responses of insects to these anthropogenic contaminants. They reported that after exposure to these compounds, there was a decrease in locomotion, exploratory behavior, and antioxidant enzymes, along with an increase in oxidative–nitrosative and inflammatory stress. *N. cinerea* has also been used to assess the toxicity of organophosphates (insecticides), specifically chlorpyrifos, a pesticide that has been banned due to its toxicity in a significant number of European countries. Nevertheless, it is the cause of the highest number of notifications for pesticide contamination in food within the Rapid Alert System for Food and Feed (RASFF). An acute injection of chlorpyrifos induced in *N. cinerea* both behavioral symptoms (such as tremors and seizures) and biochemical alterations (notably AChE inhibition) that resemble those seen in vertebrates. Moreover, administering pralidoxime 10 min after chlorpyrifos exposure effectively reversed both the biochemical and behavioral effects of the toxin [61]. The authors showed that the cockroach serves as a useful and promising insect model for preliminary in vivo studies on the therapeutic potential of new compounds against organophosphorus poisoning. Also, Santos et al. [62] used the cockroach *N. cinerea* to evaluate the acute effects of 2-borneol, and they proposed this animal model to evaluate toxicity and oxidative stress mechanisms in bioactive compounds. Moreover, cockroaches have also been used to assess the alteration of the antioxidant defense system after exposure to 4-vinylcyclohexane [63] and polycyclic aromatic hydrocarbon fluoranthene [48], and to evaluate neurotoxicity associated with caffeine [61], organophosphates [58,64], neonicotinoids [65] and pharmaceuticals [60].

Findley et al. [66] conducted a study to evaluate the pharmacological and toxicological effects of methamphetamine in cockroaches with the aim of using this animal model for future studies on substance use disorders [66].

#### Toxicology Studies Using *Gromphadorhina portentosa* as an Alternative Animal Model

*G. portentosa* stands out as a versatile model due to its tolerance to prolonged exposures, ease of observation and handling, and robust physiology, which allows for various administration routes (oral, topical, injection and indirect airborne) without compromising experimental viability [12,22,60].

Kanabar et al. [22] conducted a subchronic study lasting 26 to 60 days to evaluate the effects of dietary exposure to the herbicide Roundup Ready-to-Use III (a 2% glyphosate formulation as isopropylamine salt). Although no significant changes in survival or body weight were observed, a 32% decrease in action potential conduction velocity in the ventral nerve cord, a 29% increase in respiratory rate, and a 74.4% reduction in time spent on a motorized exercise wheel were recorded. These findings suggest that Roundup exposure may negatively impact nerve function and locomotor behavior in *G. portentosa*.

Sawczyn et al. [67] investigated whether the insecticides imidacloprid and fenitrothion, at the concentrations of 5, 10, 25, and 50 mg/kg^−1^ dry food, affected carbohydrate metabolism and nutrient absorption. Results showed that fenitrothion significantly reduced trehalose levels in insect hemolymph at all tested food concentrations compared to controls and imidacloprid produced similar effects, except at 10 mg/kg of dry food, where trehalose levels remained comparable to control values. Changes in fat body glycogen were less evident and occurred only at 5 and 10 mg/kg of imidacloprid. Overall, alterations in sugar distribution and increased glucose absorption in the midgut suggest that insects increase energy metabolism to cope with insecticide-induced stress and indicate that midgut glucose absorption parameters may serve as a non-specific biomarker of insecticide toxicity.

Taken together, these studies reinforce the usefulness of *G. portentosa* as a sensitive and versatile model organism for pesticides’ toxicological evaluation, both from a neurobehavioral and ecotoxicological perspective, including cellular, biochemical, and microbiotic levels.

### 4.4. Antibiotics and Dysbiosis

The German cockroach (*Blattella germanica*) has been used in various studies to assess the effects of antibiotics on the gut microbiota [20,55,56]. Marín-Miret et al. [56] exposed *B. germanica* to the antibiotic kanamycin in three periodic pulses. These authors observed loss of bacterial diversity and a reduction in essential symbiotic populations. The gut microbiota recovered faster after antibiotic treatments, showing its adaptation to repeated treatments. This coincided with what was observed by Li et al. [55], who reported a decrease in bacterial diversity and abundance, along with changes in the composition of the gut microbiota after exposing *B. germanica* to the ingestion of antibiotics (levofloxacin and gentamicin). Furthermore, after 14 days without antibiotic exposure, they observed that the bacterial count fully recovered, although a large portion was antibiotic-resistant. Broderick and Lemaitre [68] highlighted how the microbial composition of the insect gut can be altered by external factors such as antibiotics. Although these studies were not conducted on *G. portentosa*, they support the usefulness of invertebrate models in intestinal toxicology. The anatomical and functional homology of the digestive system with *G. portentosa* suggests it could be used as an experimental model for studying the effects on the gut microbiota following exposure to antibiotics and other xenobiotics. This is a topic of particular importance since research related to the development of antibiotic resistance is a priority in our society, and global strategies are being implemented today, such as the European Union Action Plan on Antimicrobial Resistance “One Health”.

### 4.5. Other Studies Using Cockroaches as an Animal Model

Cockroaches have a broad spectrum of applications in several other research fields. They have been used as an animal model to evaluate the influence of exposure to a low-frequency electromagnetic field [69]. These insects have also been used as models both to advance robotics research and to serve as experimental systems for studying neuroethology [13]. Marzullo [70] compared limb regeneration following autotomy versus transverse amputation, revealing clear differences in both the rate and the final size of the regenerated appendage. Moreover, they are also currently considered potentially beneficial given that medically useful compounds with antimicrobial activity can be isolated from cockroaches [40,71]. They are in fact able to adapt to harmful environments and they have a well-developed immune system. According to this, potentially useful antimicrobial substances have been isolated from cockroaches [40,71]. Recently, cockroaches have gained attention also because of the rise of insect farming to produce sustainable food [72].

#### 4.5.1. Food Use

Another area of application for these species is their use as food in livestock farms. The use of insect meals from cockroaches emerges as a highly effective and sustainable alternative to traditional protein sources like soybean meal and fishmeal [73,74,75].

In poultry, Mustafa et al. [73] significantly improved the Feed Conversion Ratio (FCR) and daily weight gain by replacing up to 12% of soybean meals with insect meals. More importantly, this enhanced the gut morphometry (increasing villus height), which allows for more efficient nutrient absorption. Similar results were observed by Ou et al. [74], who identified *P. americana* residue, a byproduct of pharmaceutical manufacturing, as a high-quality protein source for Three-yellow chickens. Furthermore, their research demonstrated that this dietary inclusion significantly enhanced digestive enzyme activity, thereby facilitating a more efficient breakdown and assimilation of fats, carbohydrates, and proteins. In aquaculture, Huang et al. [75] proved that growth rates can be maintained while significantly boosting the immune system by replacing fishmeal with *P. americana* meal. The fish showed higher levels of antioxidant enzymes (SOD, CAT) and a reduction in pro-inflammatory markers (TNF-α), indicating a stronger resistance to stress and disease.

In this context, cockroaches are included for their ability to serve as a protein base in the diet of reptiles, amphibians, or small mammals [76,77,78].

##### *Gromphadorina portentosa* Used as Food

Carvalho et al. [79,80] evaluated the inclusion of Madagascar cockroach (*G. portentosa*) meal in the diet of cockatiel (*Nymphicus hollandicus*) in captivity, focusing on chick development and adult reproductive performance. While cockroach meals do not significantly change physical growth parameters, such as body weight or the length of beaks, wings, or tails, they provide measurable reproductive benefits, such as faster recovery between laying cycles and improved hatching rates.

In another study, Garcia Perez et al. [81] investigated the potential of Madagascar cockroach meal tested at five different concentrations (0%, 25%, 50%, 75%, and 100%) as a sustainable replacement for fishmeal in the diets of juvenile Nile tilapia (*Oreochromis niloticus*). Research demonstrates that replacing fishmeal with cockroach meal had no negative impact on fish growth. Using stable isotope analysis, researchers found that nitrogen from the cockroach meal was efficiently incorporated into the fish muscle.

These studies demonstrate that Madagascar cockroach meal serves as a sustainable and highly effective alternative to traditional animal proteins whether utilized as a dietary supplement for avian species or as a primary protein source in aquaculture.

## 5. Ethical Considerations and the Application of the 3Rs to Invertebrate Research

Although invertebrates, including cockroaches, fall outside most national and institutional regulatory frameworks, researchers still bear an ethical responsibility to minimize potential harm. The principles of the 3Rs have become the cornerstone of humane experimental practice and are embedded in major regulatory instruments such as Directive 2010/63/EU [5]. While this directive applies only to vertebrates and cephalopods, several authors and organizations have argued that the precautionary principle justifies extending the 3Rs to invertebrate species whenever feasible [16,82].

Replacement. Cockroaches can serve as an alternative to vertebrate models in bioscience research. Their use may reduce reliance on vertebrates when the scientific objectives can be met with invertebrate systems. Replacement also applies within invertebrate research: computational models, cell cultures, or species with lower likelihood of sentience should be considered when appropriate.

Reduction. Even in the absence of legal oversight, experimental design should aim to minimize the number of invertebrates used while maintaining statistical validity. Transparent reporting of variability and methodology contributes to preventing unnecessary duplication of experiments.

Refinement. Refinement is particularly important for invertebrates because welfare-relevant indicators are less well understood. Nevertheless, several measures can reduce potential harm: minimizing invasive procedures or using non-invasive endpoints when possible; providing appropriate environmental conditions (temperature, humidity, shelter, substrate) to reduce stress; avoiding unnecessary handling or restraint; and using the most humane available methods for analgesia, anesthesia and euthanasia, acknowledging that evidence for optimal protocols in cockroaches remains limited [16,17].

As the evidence base grows, refinement strategies should be updated to reflect emerging knowledge on nociception, stress responses, and species-specific needs. Voluntary adoption of the 3Rs therefore provides a robust ethical framework for invertebrate research, even in the absence of formal regulation.

### Evidence for Pain and Sentience in Blattodea

The question of whether cockroaches experience pain in a subjective sense remains unresolved, but recent work highlights several features that warrant precaution. Gibbons et al. [83] reviewed the neurobiological, behavioral, and evolutionary evidence relevant to pain in Blattodea. Cockroaches possess well-developed nociceptive pathways capable of detecting noxious mechanical, thermal, and chemical stimuli. These pathways trigger rapid defensive responses and can be modulated by descending neural circuits, a feature often associated with more complex affective processing.

Experimental work in cockroaches further strengthens this view. Dessì et al. [16] demonstrated that *G. portentosa* exhibits robust nocifensive responses to thermal stimuli, including agitation, escape attempts, leg lifting, and even hissing—behaviors the authors describe as occurring “in dangerous conditions”. Importantly, these nocifensive behaviors were pharmacologically modulated by gabapentin, a drug with well-established antinociceptive effects in vertebrates.

Gibbons et al. [83] emphasize that, although definitive proof of pain is lacking, the combination of nociceptive complexity, behavioral plasticity, and evolutionary continuity supports a precautionary approach. This perspective aligns with broader discussions on invertebrate sentience and reinforces the need for welfare-oriented practices when working with cockroaches.

## 6. Conclusions and Future Directions

Cockroaches represent an alternative model of growing interest in bioscience research. Their morphology, longevity, environmental resilience, and ease of handling make them a valuable tool, especially in studies that do not require advanced technological resources or phylogenetic homologies with mammals. Their most established applications include studies in physiology, monitoring of immunological biomarkers in hemolymph, or evaluation of neurotoxic effects induced by pollutants; these insects are also gaining attention as a highly efficient and sustainable protein source, an aspect that underscores their potential in nutritional science. Additionally, cockroaches allow reproducible experimental designs, without specialized facilities, favoring their inclusion in educational settings and preliminary testing. Their use also contributes to the effective implementation of the 3R principles, contributing to reducing the use of vertebrate in experimentation. While cockroaches remain outside most formal regulatory frameworks, their use in research should be guided by precautionary principles and the 3Rs. The growing evidence that some invertebrates may experience pain-like states underscores the importance of minimizing potential harm and adopting refinement strategies whenever possible. At the same time, many aspects of cockroach biology relevant to welfare—optimal housing, humane endpoints, stress indicators, and effective anesthesia—remain insufficiently studied. Building this evidence base will be essential for developing species-specific, evidence-based care and use guidelines comparable to those available for vertebrates.

Despite their multiple experimental advantages, cockroaches present notable limitations that constrain their potential as a reference model in bioscience research, particularly in biomedical and toxicological studies. Their utility is restricted by the absence of validated regulatory protocols such as those for toxicity or ecotoxicity testing, which limits its incorporation into preclinical studies for regulatory purposes. Although they share fundamental cellular mechanisms with humans, such as neurotransmission, innate immune responses, and oxidative stress, their considerable phylogenetic distance and lack of homologous complex organs and adaptive immunity make them unsuitable for translational clinical analyses.

It is important to emphasize that the adoption of invertebrate models such as cockroaches should not be regarded as direct substitutes for vertebrate or genetically modifiable models, but rather as complementary systems Their consolidation as standardized models will depend on the generation of comparable data, validation of specific scales, and institutional recognition. Promoting their use through protocol standardization, development of specialized experimental lines, and regulatory adaptation for insect models could establish cockroaches as a reliable, ethical, and scientifically robust organism model in the coming decade.

## Data Availability

No new data were created or analyzed in this study.
